# The Role of Light Quality in Regulating Early Seedling Development

**DOI:** 10.3390/plants12142746

**Published:** 2023-07-24

**Authors:** Yunmin Wei, Shuwei Wang, Dashi Yu

**Affiliations:** 1 College of Life Sciences and Oceanography, Shenzhen University, Shenzhen 518060, China; weiym1024@163.com (Y.W.); shuweiwang_21@163.com (S.W.); 2 College of Optoelectronic Engineering, Shenzhen University, Shenzhen 518060, China

**Keywords:** light quality, photomorphogenesis, seed germination, seedling de−etiolation, shoot–root development, leaf development, greenhouse horticulture

## Abstract

It is well−established that plants are sessile and photoautotrophic organisms that rely on light throughout their entire life cycle. Light quality (spectral composition) is especially important as it provides energy for photosynthesis and influences signaling pathways that regulate plant development in the complex process of photomorphogenesis. During previous years, significant progress has been made in light quality’s physiological and biochemical effects on crops. However, understanding how light quality modulates plant growth and development remains a complex challenge. In this review, we provide an overview of the role of light quality in regulating the early development of plants, encompassing processes such as seed germination, seedling de−etiolation, and seedling establishment. These insights can be harnessed to improve production planning and crop quality by producing high−quality seedlings in plant factories and improving the theoretical framework for modern agriculture.

## 1. Introduction

It is widely acknowledged that plants are sessile and photoautotrophic organisms. Light regulates plant physiology through two major functions throughout the plant life cycle [1]. The assimilative function of light provides the energy necessary for photosynthesis, suggesting light is the ultimate energy source for green plant metabolism, and the photosynthetic efficiency depends on the spectral wavelength (light quality) [2,3,4]. On the other hand, the signaling function of light activates and regulates many key signaling pathways related to plant photomorphogenesis [5,6,7,8,9,10,11]. In developmental biology, the response of plant growth patterns to light spectra is known as photomorphogenesis, which occurs during seed germination, seedling development, and the transition from vegetative to anthesis (photoperiodic phenomenon) [12,13,14]. For instance, one seminal study showed that red light promotes the germination of lettuce seeds (*Lactuca sativa* L.) [15]. Recent studies on *Arabidopsis* have similarly shown that the process of seed germination is regulated by phytochrome B (PhyB), the primary photoreceptor involved in red−light−induced germination [9]. After germination, the seedlings exhibited an etiolated growth pattern wherein the hypocotyls were elongated and the cotyledons folded to form a hook−like structure [16]. In addition, light quality plays a crucial role in regulating plant photomorphogenesis during seedling de−etiolation [17]. Moreover, light quality also plays an important role in the transition of plants from vegetative to reproductive growth [5] and senescence [13].

**Figure 1:**
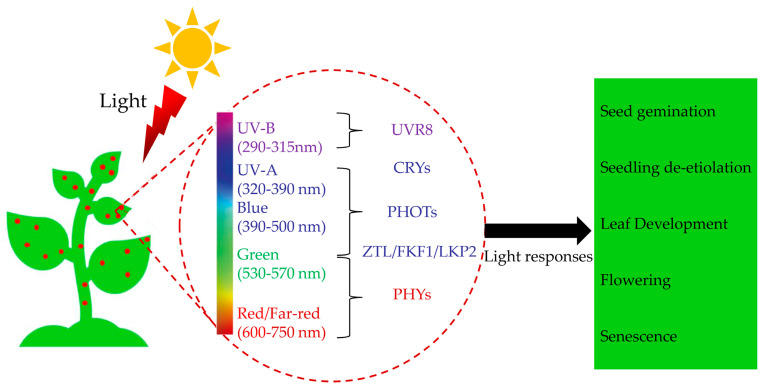
A schematic diagram depicting the involvement of light in different stages of photomorphogenesis. UV resistance Locus 8 (UVR8), cryptochromes (CRYs), phototropins (PHOTs), Zeitlupe family proteins (ZTL/FKF1/LKP2), and phytochromes (PHYs).

There is an increasing consensus suggesting that plants have evolved an array of photoreceptors that function to transduce light cues into biological signals [9,13,18,19,20]. Ample evidence substantiates that the biochemical and physiological features of five classes of photoreceptors in plants have been characterized [21,22]. It has been reported that blue and ultraviolet (UV)−A light (320–500 nm) is absorbed by three distinct classes of photoreceptors, including cryptochromes (CRYs) [23], FLAVIN−BINDING, KELCH REPEAT, F−BOX1 (FKF1), ZEITLUPE (ZTL)/LOV KELCH PROTEIN2 (LKP2) [24], and phototropins (PHOTs) [25]. Red and Far−Red light (600–750 nm) are primarily perceived by phytochromes (PHYs) and are involved in many photo−regulatory processes [13]. It has been reported that UVRESISTANCE LOCUS8 (UVR8) could sense UV−B light (280–315 nm) [26]. Overall, these photoreceptors, which perceive different light qualities, have crucial functions throughout the life cycle of plants, starting from seed dormancy and germination to seedling de−etiolation [21,22], flowering [5], and senescence [13] (Figure 1).

Raising and transplanting seedlings is commonly used in agriculture for cultivating vegetables and economic crops. Thus, producing high−quality seedlings is crucial for efficient and successful plant cultivation, providing numerous benefits, including control over growing conditions, disease and pest management, plant breeding opportunities, efficient land use, transplanting flexibility, and precise timing for planting and harvesting [27]. Seedling responses to different wavelengths can have independent effects on light−regulated development [28]. The application of light−emitting diode (LED) light systems in plant factory settings for seedling cultivation has attracted increasing attention, given their ability to flexibly control the spectral composition of light [29,30,31,32]. Therefore, it is of great significance to deeply understand how light quality regulates the growth and development of plant seedlings. Numerous studies and reviews have detailed the effects of light quality on regulating plant growth and development [21,28,33,34,35]. However, few reviews have hitherto assessed the role of light quality on young seedling development. In this review, we summarize the role of light quality in regulating early plant development, including seed germination, seedling de−etiolation, and seedling establishment, providing the foothold to refine production planning and crop quality by producing high−quality seedlings in plant factories, and a theoretical basis for modern agriculture.

## 2. The Light Signaling Pathway

**Figure 2:**
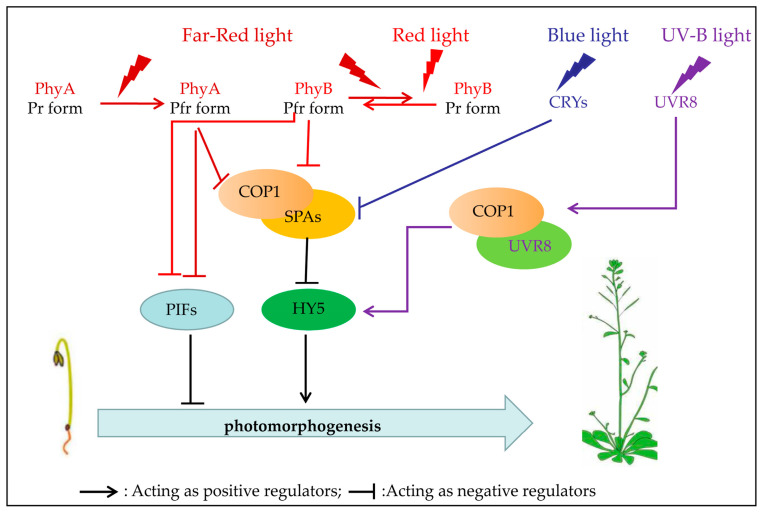
Light−mediated signaling network in regulating seed germination. Under Far−Red light, PhyA Pr in the cytoplasm converts into its active form (PhyA Pfr) and translocates to the nucleus, stimulating the degradation of COP1/SPA1 (constitutive photomorphogenic protein 1, suppressor of PhyA−105), causing the accumulation of HY5 or by facilitating the degradation of PIFs to promote photomorphogenesis. Under red light, PhyB is activated and converts to its active Pfr form, moves to the nucleus, and interacts with SPA1, preventing the formation of the COP1/SPA complex, resulting in stabilization of HY5 and up−regulates the expression of photomorphogenic genes or by mediating PIFs protein levels. The active PhyB can be reversed to the inactive form by irradiating with low red/far−red lights. Under blue light, CRYs also regulate photomorphogenesis through the COP1, SPA, and HY5 pathway. Under UV light, cytoplasmic UVR8 senses the light and binds with COP1, moves to the nucleus then stabilizes the HY5 to promote photomorphogenesis.

The light signaling pathway plays a crucial role in plant growth, development, and adaptation to their environment. Specialized photoreceptor molecules allow them to perceive light, the transformation of light signals into biochemical changes, and subsequent regulation of various physiological and developmental responses [33]. These photoreceptors can be categorized into five classes based on the wavelength of light they absorb (Figure 1) [21,22,33]. In addition, these photoreceptors further transmit the signal through a cascade to modulate the expression of multiple genes that ultimately lead to physiological responses (Figure 2).

### 2.1. Red and Far−Red Light Pathway

PHYs were the first reported photoreceptor proteins discovered in plants that enable the detection of Red and Far−Red light [13,36,37]. PHYs are evolutionarily conserved from bryophytes to angiosperms (except in the chlorophytes). In dicotyledonous plants such as *Arabidopsis thaliana*, five Phys encoded by small gene families have been identified: PhyA, PhyB, PhyC, PhyD, and PhyE [37,38]. However, in monocots, the phytochrome family consists of three members: PhyA, PhyB, and PhyC [36,39]. Furthermore, based on their stability in light, these phytochromes can be classified into photostable type I (PhyA is the only type I phytochrome) and photostable type II (PhyB to PhyE) [37,40,41]. It has been established that PhyA plays a dominant role in Far−Red light, while PhyB to PhyE regulate Red light signaling [36,41]. 

Current evidence suggests that PHYs exist in Pfr (active form) and Pr (inactive form) forms, which are interconvertible. When a PHY molecule absorbs Red light, it is converted from Pr to Pfr; when it absorbs Far−Red light, it is converted back to Pr [7,41]. This reversible conversion between Pr and Pfr serves as major molecular switches in the PHY signaling pathway, and their pathway and downstream components have been extensively studied. First, PHYs are transferred from the cytoplasm to the nucleus through the light−activated Pfr form to interact with transcription factors to regulate the expression of related genes, among which PIFs (Phytochrome−Interacting Factors) are major transcription factors that interact with PHYs [42,43]. PIFs represent transcription factors of the bHLH (basic helix–loop–helix) family that primarily function as negative regulators of photomorphogenesis [42,43]. Current evidence suggests that PhyB and PhyA interact with PIF1 to inhibit light−dependent seed germination [44]. In addition, the regulation of ubiquitin−mediated protein degradation by PHYs is an important component of the PHY signaling machinery [33]. The COP1−SPA E3 ligase complex is a regulator that plays a central role downstream of various photoreceptors [45,46,47]. It targets several positive regulators of photomorphogenesis, such as HY5 (Elongated−Hypocotyl 5), leading to its destabilization and degradation in the dark through the 26S proteasome pathway [45,48]. In summary, PHYs initiate light signaling pathways through two major negative regulators: interacting with PIF transcription factors and regulating the stability of COP1−SPA complex protein [39,40,41].

### 2.2. Blue/UV−A Light Pathway

Blue /UV−A light (320–500 nm) is mainly absorbed by three distinct types of photoreceptors, including PHOTs [23], the ZTL/FKF1/LKP2 family [24], and CRYs [25].

PHOTs are a class of protein kinases that contains serine/threonine domains and FMN (flavin−mononucleotide) binding LOV (light−oxygen−voltage) domains and are widely found in green plants [40,49]. They have been identified in ferns and mosses in addition to higher plants such as *Arabidopsis*, and their physiological functions are conserved across different species [33,49]. The signaling pathway of PHOTs begins with the absorption of Blue light and is primarily involved in mediating phototropism, which is responsible for the directional growth or movement of plants in response to light [40,49]. In addition, PHOTs also regulate other Blue−light−mediated processes, including the control of chloroplast movement, stomatal opening, and leaf expansion [40]. 

The ZTL/FKF1/LKP2 family is another type of Blue light receptor, and also has the LOV domain [24,40]. It has been shown that ZTL/FKF1/LKP2 family proteins play critical roles in integrating light and circadian signaling pathways to regulate plant development, including flowering time and the circadian clock, by controlling the stability of key light regulatory proteins [24,50]. 

CRYs are important photosensory receptors that absorb Blue/UV−A light [51]. Exposure to Blue light causes rapid phosphorylation of the cryptochrome molecule, an essential modification for its function. CRYs regulate important physiological processes throughout the plant life cycle, such as seedling photomorphogenesis, photoperiodic flowering, and circadian rhythm. Three CRYs have been identified, homologous CRY1 and CRY2 from the same family, and CRY3 from a distinct family, whose function remains to be determined [23,51]. For instance, CRY1 is involved in Blue light activation of the photomorphogenesis pathway, leading to the inhibition of hypocotyl growth, promotion of cotyledon expansion, and stimulation of chloroplast development in *Arabidopsis* [51,52,53]. 

CRYs interact with the E3 ubiquitin ligase COP1 protein, forming a complex that regulates light−dependent protein degradation [54,55]. COP1 suppresses photomorphogenic development in the dark by targeting transcription factors, including the bZIP protein HY5, for degradation. Upon light activation, CRYs bind to COP1, preventing its interaction with transcription factors, thus promoting photomorphogenesis. In addition, CRYs interact with many other proteins, but the functional significance of these interactions remains unclear. Numerous studies have demonstrated interactions between CRYs and PHYs [54,56].

### 2.3. UV−B Light Pathway

The UVR8 protein acts as the primary UV−B photoreceptor in plants and triggers a signaling cascade upon UV−B perception [18,26]. UVR8 is a homodimeric protein composed of two identical subunits. The absorption of UV−B photons by UVR8 leads to changes in the protein conformation, which result in the dissociation of the UVR8 homodimer into monomers [26,57]. After dissociation, UVR8 monomers interact with the COP1 protein, forming a complex. This interaction prevents the degradation of UVR8 and allows it to accumulate in the nucleus. Once in the nucleus, the UVR8−COP1 complex regulates the expression of various genes involved in UV−B responses. It interacts with transcription factors, such as HY5, to activate or repress gene expression, leading to the induction of protective responses, such as the synthesis of UV−absorbing compounds and DNA repair enzymes [18,58]. Plants integrate UVR8 signaling with other light signaling pathways, including the PHY and CRY pathways [28]. This interplay between pathways enables plants to synchronize their responses to diverse light wavelengths and environmental conditions.

In general, the fundamental mechanism of transducing light signals in photoreceptor𠈒mediated pathways involves direct interactions between photoreceptors and their target proteins. These interactions can occur in a light−dependent or independent manner and regulate various aspects of physiological and developmental processes controlled by light−related genes in plants [43,45,48,59]. In addition, the phytohormone pathways, including primary plant hormones such as auxin, abscisic acid (ABA), and gibberellins (GAs), are reportedly involved in plant photomorphogenesis processes [16,28,35].

## 3. Roles of Light Quality in the Regulation of Seed Germination

### 3.1. Hormones Critical for Seed Germination

**Figure 3:**
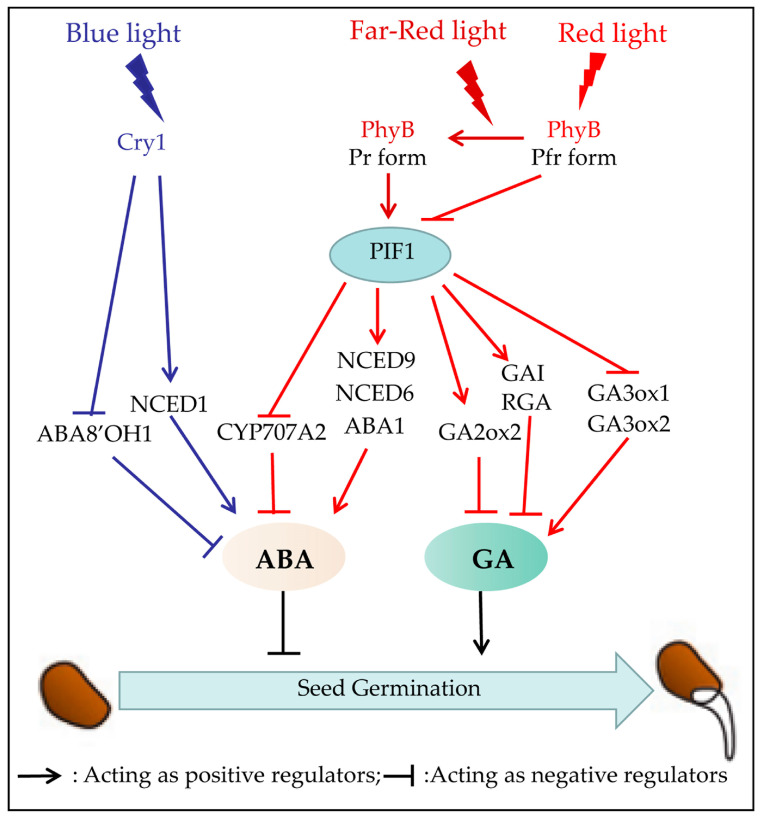
Light−mediated signaling network in regulating seed germination. Blue light suppresses monocot seed germination by enhancing the expression of the ABA biosynthetic gene, *NCED1*, and repressing the expression of *ABA8′OH1*, the ABA catabolic gene, to regulate the content of ABA in embryos. Under Red light, PhyB is activated and converted to the active Pfr form, translocates to the nucleus, and mediates the degradation of PIF1. Since PIF1 represses the expression of GA biosynthetic genes *GA3ox1* and *GA3ox2*, while activating the expression of GA catabolic gene *GA2ox2*, the degradation of PIF1 leads to increased GA levels to promote seed germination. PIF1 also induces the transcription of three ABA biosynthetic genes: *ABA1*, *NCED6*, and *NCED9*, but inhibits the expression of *CYP707A2*, which is the ABA catabolic gene. Therefore, PhyB−mediated degradation of PIF1 reduces ABA accumulation and relieves the inhibitory effect of ABA on seed germination. However, under low Red/Far−Red ratio conditions, the PhyB Pfr form converts back to PhyB Pr, allowing PIF1 to accumulate in the cell nucleus, resulting in a decrease in GA levels and inhibition of seed germination.

The effect of light quality in regulating seedling establishment commences with seed germination. It has long been established that the regulation of GA and ABA hormones is required for seed germination [22,35]. Over the years, the roles of ABA and GA in seed germination have been extensively reviewed [22,35,60,61]. ABA acts as a dormancy−inducing hormone, inhibiting seed germination under unfavorable conditions, while GA promotes seed germination by mobilizing stored nutrients, activating enzymes, and overcoming ABA−induced dormancy. The balance between these two hormones is critical for regulating seed germination and ensuring successful plant establishment [16,21,35,62]. Light quality involved in seed germination is mediated by photoreceptors [13,21] and the levels of ABA and GA, which have antagonistic functions [22,63] (Figure 3).

### 3.2. Blue Light Regulates Hormones during Seed Germination

Previous studies have identified the role of Blue light in the inhibition of seed germination, especially in cultivated cereals, including wheat (*Triticum aestivum*) and barley (*Hordeum vulgare*) [52,64]. In barley, Blue light inhibition of grain germination is dependent on CRY1, given that in germinating *CRY1a/b* RNAi seeds, Blue light results in the down−regulation of the expression of ABA biosynthetic gene *NCED1* (9−*cis*−Epoxycarotenoid Dioxygenase1), and up−regulation of the expression of ABA catabolic gene *ABA8′OH1* [52]. Growing evidence suggests that Blue light enhances the expression of *NCED1* dependent on CRY1, which increases ABA content and inhibits seed germination in dormant barley [21,64]. Extensive literature substantiates that Blue light inhibits the germination of other monocotyledonous seeds, such as in imbibed annual ryegrass (*Lolium rigidum*), wheat grain, wild grain (*Brachypodium disachyon*), etc. [20,65,66,67]. In summary, Blue light suppresses monocot seed germination by enhancing the expression of NCED1 and repressing the expression of ABA8′OH1 to regulate the content of ABA in embryos (Figure 3) [52,61,64]. The role of Blue light is mainly discussed in the context of the seed biology of monocot plants. Recent studies have reported that blue LED light could improve the rate and speed of *Stevia* seeds germination [68]. However, in dicots, the role of Blue light in seed germination and its action mechanism has not been well elucidated. 

### 3.3. Red and Far−Red Light Affects Seed Germination

The regulation of Red and Far−Red light on seed germination was discovered by *Borthwick* et al. in lettuce (*Lactuca sativa*), and the results showed that Red light−induced seed germination, whereas Far−Red light inhibited this process [15,22]. Upon exposure to Red light, PHYs are transformed into the Pfr−activated form, which promotes seed germination by controlling the content of GA and ABA by directly or indirectly regulating the expression of synthesis or metabolism−related genes. However, the conversion of Pfr to Pr in the presence of Far−Red light counteracts the effect of Red light on seed germination [16,35,69]. In dicots, from PhyA to PhyE, each member plays a distinct role in mediating seed germination in response to various environmental cues. These PHYs enable the seeds to adjust their timing and location of germination based on specific environmental signals [70]. PhyB occupies a central position in the regulation of seed germination under Red/Far−Red light irradiation, whereas phyA plays a role in mediating very low fluence responses to Red/Far−Red light [22,63]. PhyE and phyD are required for seed germination under continuous Far−Red light and very low Red/Far−Red ratios [63,70]. 

At present, the molecular mechanisms underlying PhyB−mediated germination are better understood than those of PhyA−mediated germination. It has been established that PhyB is the most crucial protein involved in initiating the early stages of seed germination. When exposed to Red light, PhyB is activated and translocated to the nucleus, facilitating the degradation of PIF1. It is well known that the PIFs negatively regulate PHY−mediated light signaling pathways by directly and indirectly regulating GA and ABA signaling [71]. However, under low Red/Far−Red ratio conditions, PhyB−induced degradation of PIF1 is dynamically reversible, allowing PIF1 to accumulate in the cell nucleus, resulting in a decrease in GA levels and inhibition of seed germination (Figure 3) [22,42].

It has been shown that PIF1 controls GA content by directly inducing the expression of two repressors of GA signaling, GAI (gibberellic acid insensitive) and RGA (repressor of *gai*−*3*) and indirectly controls GA levels by repressing the expression of *GA3ox1* (Gibberellin 3−oxidase 1) and *GA3ox2*, which are GA biosynthetic genes, while also activating the expression of the GA catabolic gene *GA2ox2* [16,72,73]. The role of PIF1 in regulating ABA levels is similar to participating in the GA signaling pathway. It induces the transcription of three ABA biosynthetic genes: *ABA1* (ABA deficient1), *NCED6* (9−*cis*−Epoxycarotenoid Dioxygenase 6), and *NCED9* (9−*cis*−Epoxycarotenoid Dioxygenase 9), but inhibits the expression of *CYP707A2*, which is the ABA catabolic gene [22,62,71]. In summary, the modulation of seed germination by Red and Far−Red light involves the interplay between phytochromes and PIF1, which regulates the ABA and GA pathways. However, there is still much to be understood in designing appropriate strategies for regulating seed germination mediated by light quality for individual plant species.

## 4. Roles of Light Quality in the Regulation of Photomorphogenesis 

### 4.1. Seedling De−Etiolation

**Figure 4:**
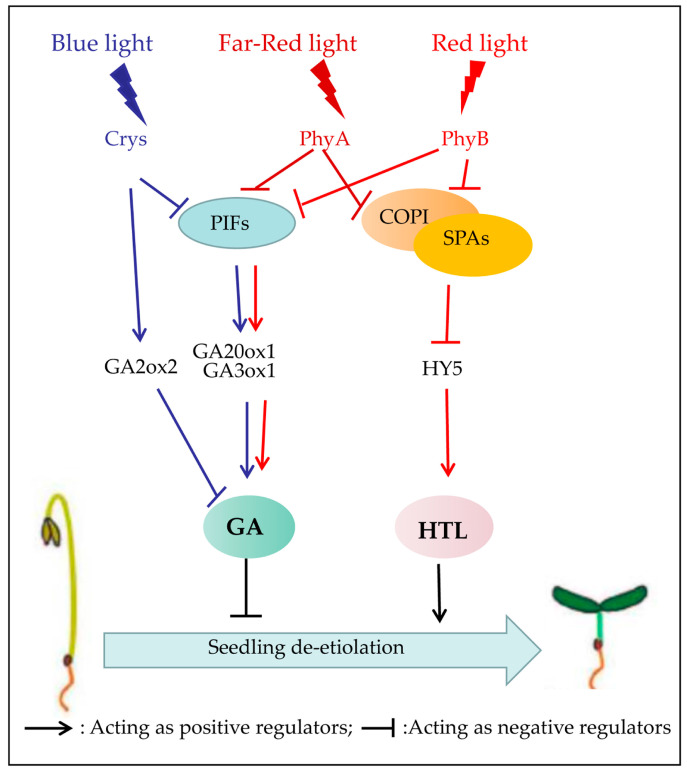
Light−mediated signaling network in regulating seedling de−etiolation. Under Blue light, CRYs repress the transcriptional activity of *PIF4*, thereby reducing the expression of downstream GA biosynthetic genes, *GA20ox1* and *GA3ox1*, while upregulating the transcript level of *GA2ox1*, a GA catabolic gene. This ultimately leads to increased GA inactivation. Under Far−Red and Red light, PHYs regulate de−etiolation events through two primary light signaling pathways: COP1/SPA−HY5 and PIFs members. HTL acts as a positive regulatory factor in the de−etiolation process.

Following germination, seedlings undergo etiolation under the soil, characterized by strong elongation of hypocotyls and closed cotyledons that lack chlorophyll and functional chloroplasts [16]. De−etiolation of the seedlings marks the major developmental switch upon their emergence from the soil as they reach the light. This phase involves the arrest of hypocotyl growth, the opening of the cotyledons, and the biosynthesis of chlorophyll, followed by chloroplast development and eventually autotrophic growth (known as photomorphogenesis) [9,13,51]. Numerous studies have revealed that Blue light and UVA induce de−etiolation mainly via CRYs, and PHYs are required for Red and Far−Red light−induced de−etiolation (Figure 4) [17,51]. 

The inhibition of hypocotyl elongation may be a characteristic phenotype in de−etiolation studies [51]. The inhibition of hypocotyl elongation in response to Blue light is mainly mediated by CRY1, for instance, the *cry1* (*hy4*) mutant weakened the inhibitory effect of Blue light on hypocotyl elongation [74]. CRYs regulate Blue−light−induced hypocotyl elongation by mediating the GA inactivation gene (*GA2ox2*) and the genes involved in GA synthesis (*GA20ox1* and *GA3ox1*) expression [75,76]. Song et al. revealed that CRYs could repress the transcription activity of PIF4 by binding to it, thereby reducing the expression of the downstream *GA20ox1* and *GA3ox1* genes and upregulating the transcript level of *GA2ox1*, leading to increased GA inactivation. These processes ultimately result in reduced levels of GA and shorter hypocotyls [75]. CRY2 is also involved in Blue light−induced de−etiolation through COP1/SPA−HY5 pathways [51]. 

PHYs play a central role in Red and Far−Red light−regulated de−etiolation events via two main light signaling pathways, COP1/SPA−HY5, and four PIFs members (PIF1, PIF3, PIF4, and PIF5) [13,16,75,77]. The COP1/SPA complex, as an E3 ubiquitin ligase, negatively regulates the levels of several photomorphogenesis−promoting proteins, such as HY5 [77,78]. Specifically, the COP1 complex with SPA1 directly interacts and ubiquitinates HY5, which directly binds to both the C/G box and G box in the promoter of HTL (hypersensitive to light) [13,46]. HTL is a positive regulatory factor of the de−etiolation response mediated by PHYs and CRYs [79]. In addition to the COP1/SPA−HY5 pathway, the PIFs also play a central role in the de−greening process of seedlings [42]. Red and Far−Red light promote photomorphogenesis due to the phosphorylation and rapid degradation of PIFs mediated by PhyA and PhyB [13,42]. UV−B also mediates seedling de−etiolation via its dependence on the UVR8 and COP1/SPA−HY5 pathway [28]. However, the mechanism of de−etiolation in monocots has not been thoroughly studied. It is worth noting that in rice, a member of the PIF family called OsPIL15 is involved in repressing etiolated seedling growth [80].

### 4.2. Shoot–Root Development 

Despite growing beneath the soil, roots in plants are still impacted by light signaling transmitted from the shoot, which can influence the development of both primary and lateral roots [81]. Several studies have shown that COP1, HY5, and UVR8 play important roles in root and shoot growth and demonstrated how light and photoreceptors regulate root and shoot growth [81,82,83,84]. In shoots, COP1 induces PIN1 (PIN−FORMED 1) transcription to regulate shoot−to−root polar auxin transport and the intracellular distribution of PIN1 and PIN2 in roots to influence growth and development [85]. HY5 is also involved in regulating root growth and development by light quality [81,86]. The reduction in lateral root density induced by the joint application of white and Far−Red light is thought to be mediated by HY5, given that *hy5* mutants exhibit a similar reduction in lateral root density compared to wild−type plants, regardless of whether they are exposed to Far−Red light or not [87]. The stability of HY5 in roots is modulated by Blue light through its interaction with CRYs, which leads to the activation of miR163 and HY5 and promotes primary root growth [88,89]. Further studies revealed that HY5 regulates lateral root emergence by affecting auxin signaling. Recent studies have reported that UVR8 directly interacts with MYB73/MYB77 (MYB domain protein73/77) transcriptional factors, to modulate shoot and root growth in *Arabidopsis* [89]. In addition to indirectly modulating root growth by regulating shoot growth, light is also directly involved in the regulation of root growth; however, the underlying mechanisms remain unclear. It is widely thought that roots and shoots must coordinate their growth responses to allow better growth of the whole plant. Therefore, further research is warranted to fully understand how light coordinates the development and growth of shoots and roots.

### 4.3. Leaf Development

The shade avoidance response (SAR) is a plant response mechanism that aims to optimize the acquisition of light energy for photosynthesis during vegetative growth. The SAR is characterized by increased hypocotyl, stem, and petiole elongation, a more erect leaf position, increased apical dominance, and early flowering [28,90]. Importantly, it is now understood that shade from vegetation has a distinct spectral signature. UV−B, F, and Blue light in unfiltered sunlight activate their corresponding photoreceptors: UVR8, PHYs, and CRYs. These photoreceptors directly or indirectly converge to inhibit PIFs and the COP1/SPA complex to regulate SAR [91]. UV−B, Red, and Blue light are depleted in shaded environments, while Far−Red light is relatively abundant. Consequently, UVR8, PHYs, and CRYs activity are greatly reduced, preventing the inhibitory action of these photoreceptors on COP1 and PIFs. Moreover, under low Red/Far−Red ratio conditions, the Pfr form converts to the Pr form, leading to re−accumulation and stabilization of PIFs, which promote stem elongation. In addition, under low Red/Far−Red ratio conditions, PIF1, PIF5, and PIF7 are involved in plant hypocotyl elongation by mediating auxin signaling [92,93]. Leaf growth and development are closely associated with SAR. Low−light environments trigger the upward positioning of leaves, which is typical of SAR and has been shown to depend on the combined action of PHYs and CRYs [94,95]. Light quality also significantly influences the development of leaf thickness [96]. Under the condition of increasing the Blue light ratio, rapeseed leaves formed two cell layer tissues on the fence to thicken the leaf thickness [97]. Blue light is also known to promote palisade cell development through phot2 [98]. Lettuce leaves increase in width and length when irradiated with green LED light of higher photosynthetic photon flux [99]. UV−B exposure also includes relatively thicker leaves, shorter petioles and leaf curling in plants [100].

### 4.4. Stomata Development

The stomata are openings on the leaf surface, which mediate gas and water vapor exchange between the plant and the environment. Numerous studies have indicated that light modulation of stomatal development is another crucial aspect directly related to plant photomorphogenesis [17,101,102]. Light plays a key role in forming mature stomata and ensuring proper stomatal patterning [103]. Consistently, several studies have shown that the formation of M (Meristemoid) and stomatal maturation is compromised in *phyB*, *phyA*, and *cry1cry2*, the photoreceptor loss−of−function mutants [54,103]. In *Arabidopsis*, the components of light signaling (such as COP1 and PIFs) and those of the stomata developmental pathway work synergistically to regulate the whole process of stomatal development [103]. In the dark, single loss−of−function mutants of *cop1*, *cop10*, and *det1* exhibited constitutive clustered stomata, suggesting that COP1 and SPA proteins all act to repress asymmetric cell division and stomatal fate initiation [54,104,105]. Recently, studies have substantiated that COP1 facilitates YDA activity to suppress stomata formation by promoting phosphorylation and subsequent degradation of SPCH (SPCHLESS) and ICE1 [47,103,106,107]. Substantial evidence also indicates that PIFs accumulate in the dark and work synergistically with the COP/SPA complex to suppress photomorphogenesis [39,103]. In *Arabidopsis*, PIF4 directly inhibits *SPCH* expression, suppressing stomatal development in response to higher temperatures [108]. Another report indicated that the PIF−GNC/GNL module is important in light−mediated stomatal development. PIFs can directly repress the expression of two paralogous genes, *GNC* and *GNL*, which could promote cell division and stomata formation in cotyledons and hypocotyls during the dark−to−light transition [109]. Although the past few years have witnessed significant scientific progress, knowledge of the regulatory mechanisms of stomatal development and patterning in cereal grasses like maize, rice, barley, and Brachypodium remains rudimentary, even though significant progress has been made in understanding the mechanisms of light−regulated stomatal development and behavior in *Arabidopsis* [103].

### 4.5. Chloroplast Development

The light signal is important for the biogenesis and development of chloroplasts, which are crucial for plant growth [110,111]. Chloroplast development is regulated jointly by nuclear and plastid genes, for example, the photosynthesis−associated nuclear−encoded genes (*PhANGs*) and the photosynthesis−associated plastid−encoded genes (*PhAPGs*). *PhANGs* are transcribed by the RNA polymerase NEP (nuclear−encoded RNA polymerase) encoded by the nuclear genome, while *PhAPGs* are transcribed by the plastid RNA polymerase PEP (plastidial RNA polymerase). It has been established that PHYs could activate these two major classes of genes by inhibiting PIFs [112,113]. Upon light activation, the PHYs induce the PEP complex to complete their assembly in the plastid, thus initiating *PhAPG* transcription. Conversely, in the dark, the PIFs can suppress the transcription of the nuclear gene *PhANGs*, and inhibit the formation of the PEP complex and the transcription of *PhAPG* in the plastid [112,113]. On the other hand, the Blue light induces the expression of the nucleus−encoded *SIG5* gene, which acts as a retrograde and light−controlled regulator of chloroplast function, and is primarily mediated by CRY1, a photoreceptor that responds to Blue light [51,114]. Therefore, both PHYs and CRYs play a pivotal role in the development of etioplasts into chloroplasts.

## 5. Roles of Light Quality on Seedling Quality

It is well known that high−quality seedlings are essential for optimal plant growth and development. Light quality has a significant effect on the quality of seedlings, including seedling growth and accumulation of organic matter. For instance, Red light benefits stem growth and stimulates hypocotyl elongation, cotyledon expansion, plant height, and leaf area in tomato [115]. In tobacco, Red light promotes lateral root formation and auxin polar transport from the shoot to root, enabling optimal root morphological development, and is critical for the successful growth of seedling transplants [116]. Combination light is more favorable for seedling growth than a single light, and among them, the combination of Red and Blue light is considered the best light quality to promote the growth of most plants [117,118]. For example, in the cultivation of cucumber seedlings, the environment of Red plus Blue light is more beneficial to the development of seedlings, plant height, and dry matter quality than the monochrome Red and Blue light treatment [118]. The combination of Blue–Red–Green light is proven to be more effective in promoting growth rate, shoot height, root length, leaf area and new root development of pomegranate seedlings [29]. Increasing the proportion of Blue light effectively reduces the plant height, and increasing the proportion of Red light allows for more photosynthetic products to be transported to the leaves of cucumber seedlings [118]. Thus, these findings will contribute to the design of more energy−efficient supplemental lighting to aid in the industrial production of high−quality seedlings.

In addition, light quality also significantly affects the carbohydrate reserves of seedlings, such as the accumulation of soluble sugars and proteins, which are important characteristics of high−quality seedlings [119]. Many studies have found that Red light increases soluble sugar levels in cucumber, radish and pea seedlings [120,121]. In tomato, Blue light significantly increases soluble sugar levels compared to other types of light, whereas the highest amount of soluble proteins was observed in Red plus Blue light conditions [122]. Research has demonstrated that supplementing Red, or Blue with white light effectively increases the soluble sugar level of hydroponic lettuce in greenhouses [123]. Taken together, Red and Blue light are more effective for increasing soluble sugars and proteins levels in seedlings, possibly because Blue and Red light are the two main types of light quality that drive photosynthetic biosynthesis.

## 6. Conclusions and Perspectives

Over the past two decades, the application and impact of light quality in horticulture have become a research hotspot [75,124,125,126]. Herein, we sought to illustrate the molecular mechanism of light quality in regulating early seedling development, including seed germination and seedling photomorphogenesis. Lights with different wavelengths have varying effects on plants at different developmental stages. Red light promotes seed germination, while Blue light and low Red/Far−Red ratio conditions inhibit seed germination. Both Red and Blue light promote the seedling de−etiolation process. Photoreceptors PHY and CRY have many intersections in downstream signals, including the regulation of seedling development, leaf development, stomatal development, and chloroplast development through the COP1/SPA and PIFs signaling network. Additionally, with the advancement of LED technology, lighting systems with various spectral ratios can be developed and applied to modern horticulture and factory production of seedlings [29,30,32]. Therefore, deeply understanding how light quality regulates the growth and development of plant seedlings is of great significance for greenhouse horticulture to utilize these insights to improve production planning and crop quality. 

Despite considerable progress in the understanding of molecular events underlying photomorphogenesis, there are still a large number of unresolved issues. Moreover, the influence of light quality on plant growth is relatively complex, and *Arabidopsis thaliana* is often used as a model plant in laboratory settings. Therefore, there is an urgent need for research to understand how knowledge gathered in *Arabidopsis* can be applied to understand the adaptation of greenhouse horticultural plants to their local environment and to improve production schedules and crop quality.

## Data Availability

No new data were created.
